# New Remedies: The Method of Preparing and Administering Them; Their Effects on the Diseased and Healthy Economy, &c.

**Published:** 1840-04

**Authors:** 


Art. XI.—1. *New Remedies: the method of preparing and administering them; their effects on the diseased and healthy economy, &c.* By Robley Dunglison, m.d., Professor of the Institutes of Medicine in Philadelphia College.—*Philadelphia*, 1839. 8vo, pp. 503.

2. *A Compendium of Materia Medica and Pharmacy, adapted to the London Pharmacopœia, embodying all the new French, American, and Indian Medicines, &c.* By J. H. Lane, m.d., &c.—*London*, 1840. 12mo, pp. 308.

3. *A Compendium of Materia Medica, Pharmacy, and Toxicology. For the Use of Medical Students.* By D. Spillan, m.d.—*London*, 1839. 12mo, pp. 80.

We class these works together, because their general subject and object are similar: but this is their only resemblance.

1. The work of Dr. Dunglison is by far the completest that has yet appeared on the subject of the additions to our therapeutic means in these latter years; and it is, like all the productions of this accurate and industrious writer, full of information of great value to the practitioner. Each substance is treated of at great length, and mostly under the following heads: Method of preparing; Effects on the economy in health; Effects on the economy in disease; Mode of administering. Under this last head, many formulæ are generally given, original or selected from the best practical authors, British, American, or continental. The copiousness with which the different articles are treated of may be judged from the number of pages occupied by a few of the best known: thus, hydrocyanic acid occupies fifteen; creosote twenty-six; iodine twenty-eight. We strongly recommend this work to the notice of practitioners. It is equally suited to British as to American practice.

2. Dr. Lane's little volume is on the same general plan as Dr. Thomson's long-known and popular Conspectus; but it is much fuller in its details, more especially in the chemical department. It seems carefully compiled, is well suited for its purpose, and cannot fail to be useful.

3. Dr. Spillan's pamphlet is little more than a tabular view of the materia medica, presenting, in distinct columns, their name, effect, uses, and doses. It is intended and is only suited for the mere student.

